# Promoting STI self‐testing through HIV self‐testing

**DOI:** 10.1002/jia2.26138

**Published:** 2023-06-26

**Authors:** Dongya Wang, Rayner Tan, Gifty Marley, Joseph D. Tucker, Weiming Tang

**Affiliations:** ^1^ University of Miami School of Communication Miami Florida USA; ^2^ Dermatology Hospital of Southern Medical University Guangzhou China; ^3^ University of North Carolina Project‐China Guangzhou China

1

HIV self‐testing (HIVST) refers to the process in which individuals collect their own specimens (e.g. blood, saliva and urine), perform the test and interpret the results at a convenient time and place [1]. HIVST has been recognized as an innovative and promising approach to increase testing uptake, expand the HIV testing rate and enhance HIV testing coverage [1, 2]. Moreover, a large body of HIVST programmes and research worldwide also proved the feasibility, acceptability and effectiveness of HIVST in HIV prevention [1, 3]. Hence, the World Health Organization (WHO) recommended HIVST as the alternative and promising way to decentralize HIV testing and further increase the uptake of HIV testing, especially in low‐ and middle‐income countries [4].

More importantly, the experiences learned from HIVST programmes can be utilized to facilitate other sexually transmitted infection (STI) prevention programmes. Although the STI epidemic has become a global public health issue, it was often overlooked and underfunded. Given the advantages of self‐testing, scholars and health practitioners proposed STI self‐testing as an alternative way to facilitate the STI testing rate and coverage outside clinical settings. However, previous self‐testing research and implementation programmes focused on HIVST or STI self‐testing separately [5], which lost the opportunity to promote HIV and STI testing simultaneously. Here, we advocate that integrating STI self‐testing with HIVST may be useful for expanding STI testing.

First, the high feasibility of HIVST has opened a pathway for STI self‐testing. HIVST is easy, convenient [6] and less stigmatized [7]. By integrating STI self‐testing with HIVST, the demand generated by HIVST can be leveraged to improve STI testing coverage. For example, a clinical trial conducted in China demonstrated that rapid dual self‐testing for HIV and syphilis expanded syphilis testing uptake among men who have sex with men (MSM) in China [8]. Studies in several other countries (e.g. the United States and Australia) also confirmed this finding. As a result, the WHO recommended the dual HIV/syphilis rapid test as the alternative option to expand HIV and syphilis testing rate [4].

Second, the integration would be more cost‐effective than HIV or STI self‐testing alone. By integrating programmes, resources can be leveraged for multiple STI testing instead of HIVST alone. Since HIVST alone is already a cost‐effective strategy for promoting HIV screening [9], integrating STI self‐testing with HIVST would be more cost‐effective. With the increasing burden of syphilis and other STIs globally, there is a strong need to promote STI testing [6]. The HIV‐STI integrated model should be affordable in diverse settings, especially in low‐ and middle‐income countries.

Third, the integrated self‐testing model can decentralize STI testing, improving the coverage of STI testing. Traditional STI testing mainly relies on clinic testing, which may be more stigmatized, centralized and hard to access for at‐risk individuals [8]. With the shifting budgets and closure of clinics due to COVID‐19 and other issues, a lack of access to STI testing may further exacerbate the STI epidemic [10]. This has been demonstrated in a study conducted in the United States [11]. To address this problem, decentralized STI testing and promoting a people‐centred STI testing strategy is essential, while integrating STI self‐testing with HIVST can empower and facilitate routine STI tests.

However, there are a few things that need to be taken into consideration before the scale‐up of an integrated HIV‐STI self‐testing model.

1. More high‐quality rapid test kits that can be used in diverse settings are needed. Many *Neisseria gonorrhoeae* (GC) and *Chlamydia trachomatis* (CT) rapid testing kits in many parts of the world have poor accuracy [12, 13], which thwarted the scale‐up of the integrated self‐testing model. Generally, those self‐testing kits may have lower sensitivity than laboratory‐based tests, which can produce false‐negative results (Table 1). Therefore, we advocate that more high‐quality rapid test kits are needed for the integrated model while acknowledging the important role of those rapid test kits in supplementing other’ screening approaches.
2. Self‐testing kits that can separate ongoing and past infections are needed to capture new infections of syphilis. This recommendation was derived from the evidence that dual HIV/syphilis self‐testing kits may not distinguish new from old infections [8]. Some kits can only identify whether the patients are ever infected rather than separating new and past infections. Given its importance, we encourage research and development focusing on integrated self‐testing kits, which can distinguish new from past infections.
3. More innovative solutions are needed to link self‐reported data with national surveillance. Integrated self‐testing might reduce the opportunities for tracing people with positive results and linking them to care [6]. Furthermore, the self‐testing relied on self‐reported data, which generated missing national data, and might lead to insufficient evidence of shifts in patterns of HIV and STIs infection [6]. To tackle this issue, we suggest the establishment of a national digital reporting platform as a promising solution to incorporate more self‐reported data into the national data repository. Individuals who undertake HIV and STI self‐testing are encouraged to share their results on this platform, thereby enabling them to keep track of their HIV and STI status while assisting the Centers for Diseases Prevention and Control (CDC) in acquiring more individual‐level data. Hence, more innovative ways can be adopted to link self‐reported and national data in terms of HIV and STI self‐testing results.

**Table 1:** Examples of performance characteristics of different STI rapid kits

Categories	Test evaluated	Specimen	References assay	Sensitivity (95% CI)	Specificity (95% CI)	Studies
HIV rapid kit	OraQuick ADVANCE Rapid HIV 1/2 Antibody Test	Oral fluid	Abbott Architect HIV Ag/Ab Combo assay	87.50%	99.70%	Neuman et al. [14]
	Abbot Determine™ HIV‐1/2 antibody test	Blood	Abbott Architect HIV Ag/Ab Combo assay	98.90%	99.60%	Lee et al. [15]
Syphilis rapid kit	Alere Determine™ Syphilis TP test	Blood	TP‐specific tests	86.32%	95.85%	Jafari et al. [16]
	Visitect Syphilis test	Blood	TP‐specific tests	74.26%	99.43%	
	SD Bioline V.3.0 test	Blood	TP‐specific tests	84.50%	97.95%	
Dual HIV/syphilis rapid kit	SD BIOLINE HIV/syphilis Duo rapid test	Blood	ELISA for HIV; TPHA for syphilis	100% for HIV; 97.6% for syphilis	99.5% for HIV; 96.0% for syphilis	Shimelis and Tadesse [17]
Chlamydia trachomatis (CT) rapid test	Acon Chlamydia Rapid Test	Vaginal swab	NAAT	66.70%	91.30%	Hurly et al. [18]
	DRW Chlamydia Rapid Test	Urine	PCR assay	47.00%	98.80%	Wisniewski et al. [12]
	QuickVue Chlamydia Rapid Test	Cervical swab	NAAT	37.70%	99.40%	Nuñez‐Forero et al. [19]
Neisseria gonorrhoeae (GC) rapid test	OneStep Gonorrhea RapiCard InstaTest	Urine swab for males; vaginal swab for females	Culture	32.40%	96.00%	Abbai et al. [13]

Abbreviations: ELISA, enzyme‐linked immunosorbent assay; NAAT, nucleic acid amplification test; PCR, polymerase chain reaction; TP, treponema pallidum; TPHA, treponema pallidum hemagglutination assay.

In summary, given the experiences obtained from HIVST programmes, we are recommending integrated HIV and STI self‐testing models to facilitate simultaneous HIV and STI testing. We propose that the integrated self‐testing kit could be the alternative approach to address this issue. Our proposal relied on a series of advantages proffered by the integrated self‐testing kit, including its high feasibility, cost‐effectiveness and the decentralization of facilitating STI tests outside of clinical settings. Despite those benefits, there are still barriers inhibiting the scale‐up of the integrated self‐testing model. Hence, to roll out STI tests with HIVST, we call for high‐quality rapid test kits on the one hand. On the other hand, we advocate conducting more empirical studies to provide substantial experience which will be beneficial for the future implementation of the integrated model.

## COMPETING INTERESTS

The authors declare no competing interests.

## AUTHORS’ CONTRIBUTIONS

DW drafted the manuscript. WT made revisions. RT, GM and JDT provided feedback on the draft and revision. All authors equally contributed to this response. All authors read and approved the final version.
